# ESTraS, an easy and simple tracking system for assessing rodent behavior

**DOI:** 10.3758/s13428-024-02537-7

**Published:** 2025-02-03

**Authors:** A. Morcuende, T. Femenía, J. Manzanares, A. Gasparyan

**Affiliations:** 1 Institute of Neurosciences, UMH-CSIC, Alicante, Spain; 2 Departamento de Farmacología, Universidad Miguel Hernandez (UMH)Avda Ramón y Cajal S/NSan Juan of Alicante, 03550 San Juan de AlicanteAlicante, Spain; 3 Redes de Investigación Cooperativa Orientada a Resultados en Salud (RETICS), Red de Investigación en Atención Primaria de Adicciones (RIAPAd), Ministerio de Ciencia E Innovación (MICINN), Instituto de Salud Carlos III, Desarrollo Regional (FEDER), Madrid, Spain; 4 Instituto de Investigación Sanitaria y Biomédica de Alicante (ISABIAL), Alicante, Spain

**Keywords:** Software, Self-coding, Rodent behavior, Data analysis by clustering

## Abstract

Animal behavior analysis software has become an essential tool in the life sciences. However, the currently available tools have some significant shortcomings that limit their use by individuals without programming skills, access to higher informatics resources, or the capability to invest large sums of money. We have developed and validated an easy-to-use and straightforward tracking system named ESTraS to address this issue. This freeware software allows researchers to track and analyze rodent behaviors, offering additional options such as trajectory and angle analysis. Through ESTraS, researchers can utilize unsupervised clustering techniques, such as *k*-means or hierarchical clustering, to further explore the obtained results. This clustering enables the classification of results based on observed similarities among subjects. The data of this manuscript shows that ESTraS can prove to be extremely valuable, not only by providing essential behavioral analysis tools but also by offering specific data analysis options with just one click.

## Introduction

The automated evaluation of rodents’ behavior is a widely used practice in life sciences. Traditional manual analyses have been performed in behavioral assessment, encompassing motor activity, anxiety, social interaction, and object recognition. However, automated systems have become a valid option, offering many benefits. Using these systems optimizes the researcher’s time and resources and mitigates bias, ultimately bolstering the reproducibility of research outcomes. The first systems developed for this purpose were commercial ones, further increasing inequalities depending on researchers’ access to such systems. Therefore, in recent years, following the philosophy of open science and accessible to all, some freeware systems have been developed (Pennington et al., 2019; Rao et al., 2019; Zhou et al., 2023). However, most require users to have basic programming skills and present limited tools for analyzing the results obtained.

To respond to these shortcomings, we have developed an easy and simple tracking system (ESTraS) as freeware software designed as a Python standalone executable (Bradski, 2000; Clark, 2015; Fabian Pedregosa et al., 2012; Fredrik, 1999; Harris et al., 2020; Hunter, 2007; McKinney, 2010; Virtanen et al., 2020; Wannes Meert, 2022; Waskom, 2021). Therefore, researchers do not need programming skills or to install any program, and they can perform all the behavioral analyses and further exploration with a single click. Even this software could be more extensive, using some simple behavioral tests and more complex ones. We have validated it using two of animal research’s most used behavioral tests: the light–dark box (LDB) and the open field (OF) tests. Even more complex behavioral tests could be evaluated based on rodents’ exploration abilities. Throughout the text, we demonstrated that this software is handy, easy to use, and entirely valid for carrying out simple or more complex behavioral studies. Notably, ESTraS allows (1) to track rodents in the behavioral apparatus, (2) to analyze several behavioral aspects provide information about motor activity (distance traveled), anxiety-like behaviors (time spent in different regions of interest (ROIs), mean speed, distance traveled in various ROIs) and (3) to provide tools for further explore the rodents’ trajectories and angles, using Dynamic Time Warping and two different clustering types (*k*-means and hierarchical clustering). To our knowledge, this is the first freeware software providing tools for further data analysis using two unsupervised clustering methods to classify subjects according to the similarities in their behavior.

## Methods

### Animals

A total of 16 male C57BL/6 J mice (6 weeks old) were purchased from Charles River laboratories (Lille, France) and were housed in groups of five per cage in the Service of Animal Facilities of the Miguel Hernandez University, under controlled environmental conditions of temperature (21 ± 2 ºC), relative humidity (60 ± 10%), and light–dark cycle (lights on from 08:00 to 20:00 h). Food and water were available *at libitum*. After 1 week of acclimatization, the experimental procedures began. All experimental procedures complied with the Spanish Royal Decree 53/2013, the Spanish Law 32/2007, and the European Union Directive of the 22nd of September 2010 (2010/63/UE) regulating the care of experimental animals and were approved by the Ethics Committee of Miguel Hernandez University.

### Behavioral evaluations

*Light–dark box test (LDB)*. It is a widely used test to assess rodent anxiety-like behaviors (Crawley & Goodwin, 1980). The test used a methacrylate apparatus with two compartments (20 × 20x20cm). One is entirely opaque, and the other is white and illuminated (600 lx), separated by a panel with an opening that allows the rodent to circulate freely. At the beginning of the test, mice were individually placed in the center of the light compartment facing the opaque one, and the recording started.

*Open field test (OF)*. Mice were placed into individual methacrylate boxes (25 × 25x25cm) and videotaped for 10 min, as we did previously (Gasparyan et al., 2021). In each session, four mice were evaluated simultaneously, the maximum number of mice the ESTraS software can analyze simultaneously.

### ESTraS software use

**Fig. 1:**
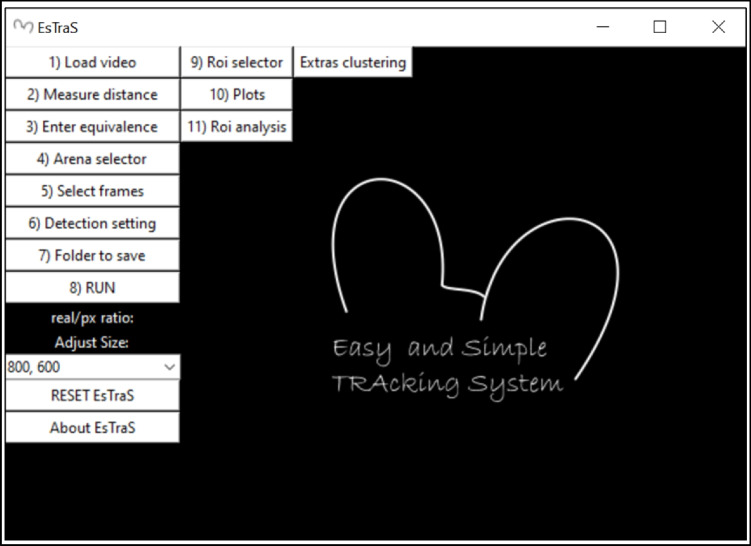
ESTraS software with different options to be clicked consecutively for easy analysis

**Fig. 2:**
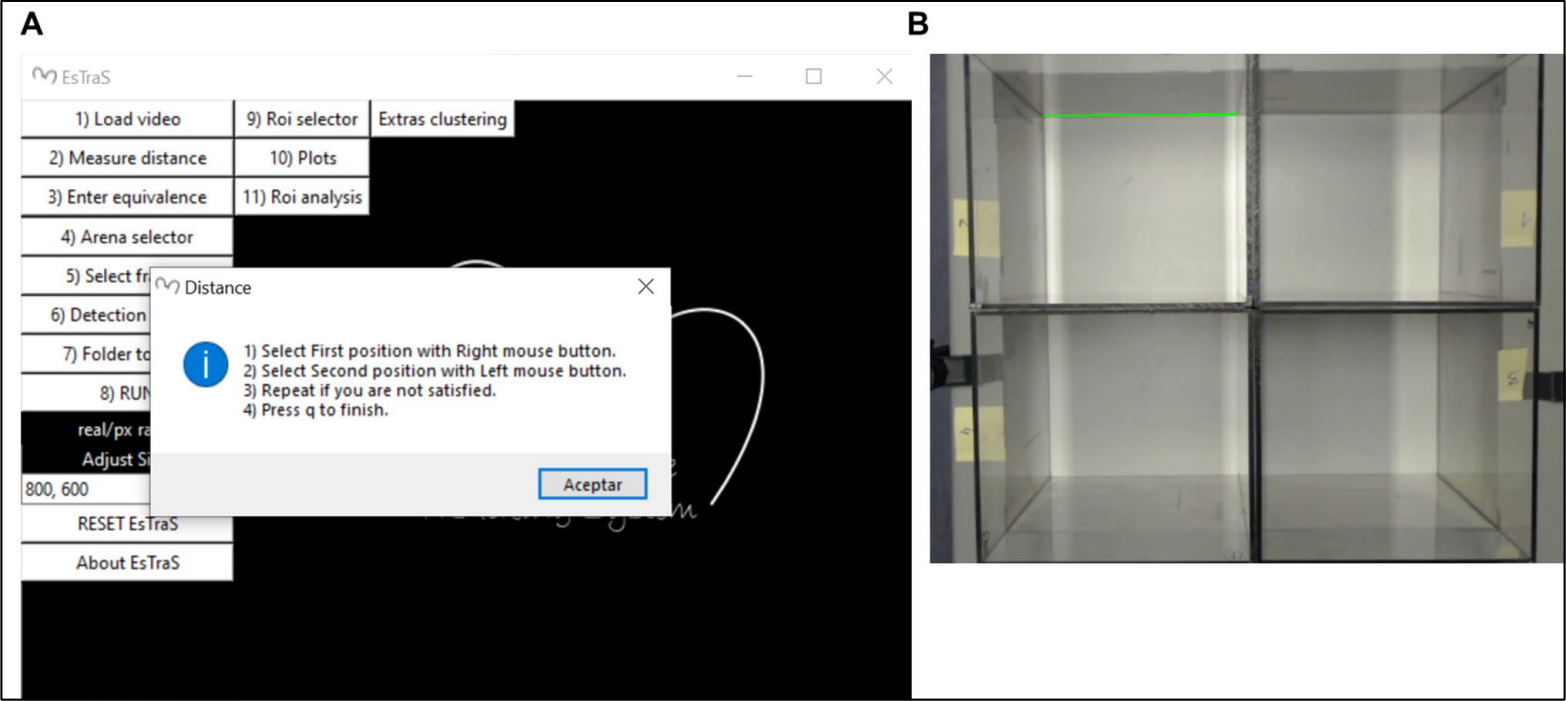
Distance measure in the ESTraS software

**Fig. 3:**
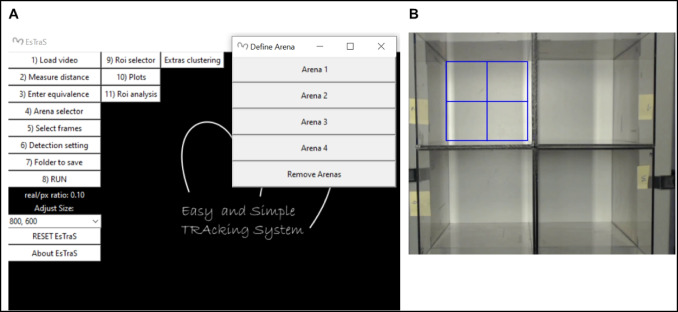
Arena selection in the ESTraS. The software has been designed to analyze up to four arenas simultaneously

**Fig. 4:**
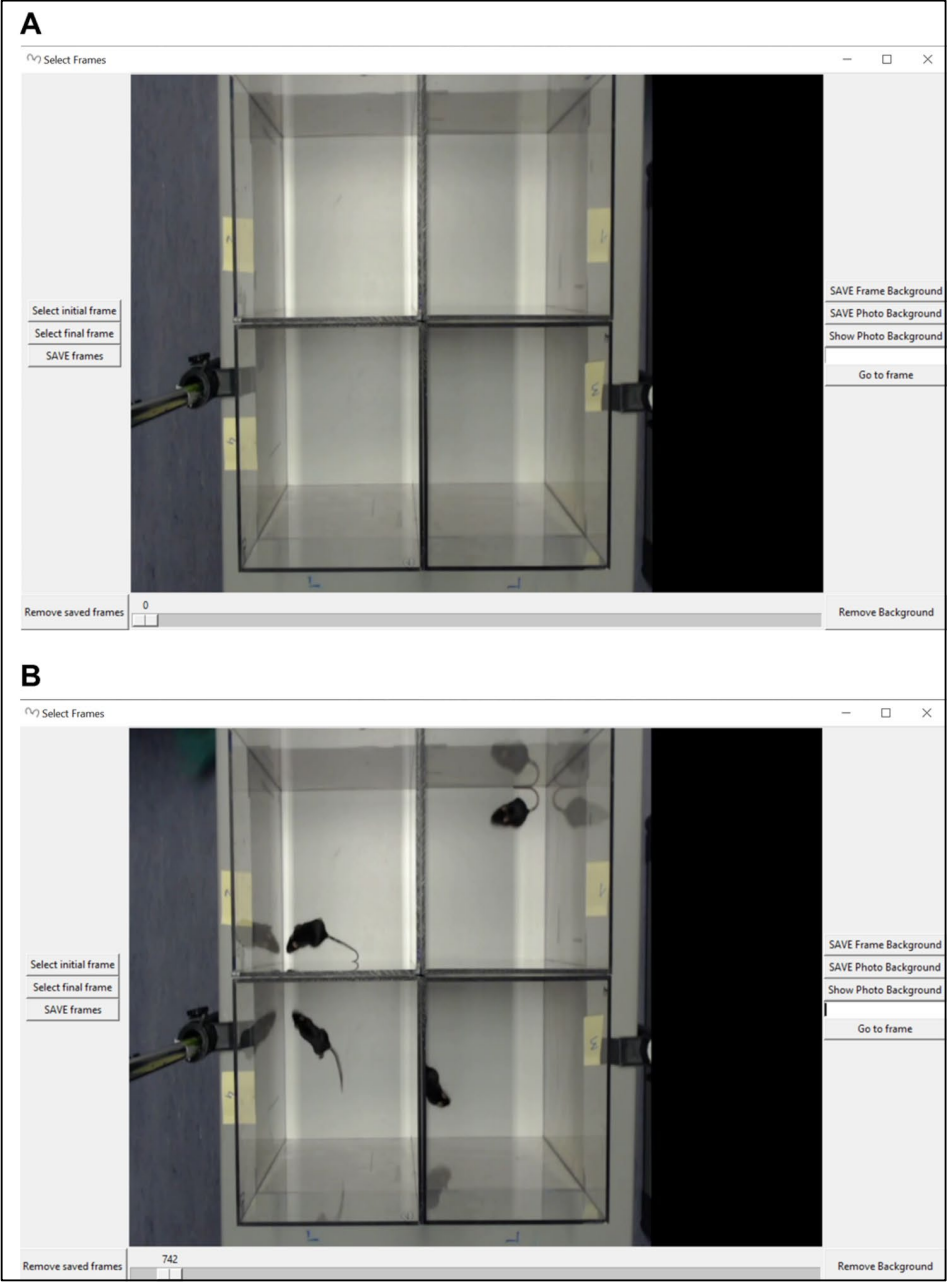
Selection of the initial and final frames for the analysis. A frame background is encouraged for correct detection

**Fig. 5:**
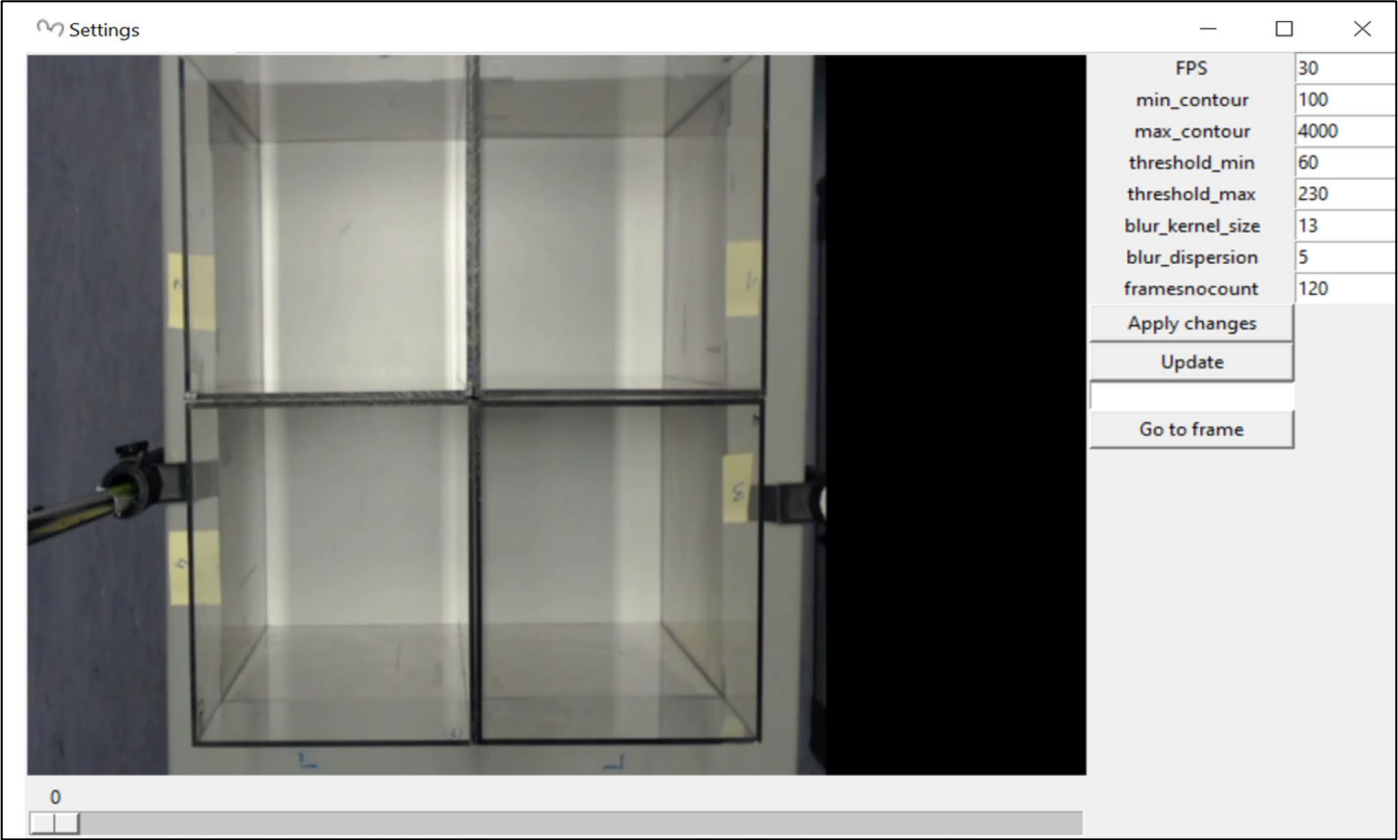
Detection settings in the ESTraS to improve object detection

After recording both tests, the analyses with ESTraS begin, comparing the results with the manual evaluation in the case of the LDB and with commercially available software (SMART Video Tracking Software, Panlab) for the OF test. To perform the corresponding evaluation, we follow these steps (also available in the handbook of the software):

- Click on the option *load video* and select the video we want to analyze (Fig. 1).
- On point *measure distance*, we followed the instructions regarding the form to select the distance and *enter equivalence* (Fig. 2).
- We selected all four arenas we used for our evaluations in the OF test by clicking on the *arena selector* (Fig. 3).
- In point number 5, we selected different tools for the correct evaluation. Firstly, we saved the *frame background* at the beginning of the test. At this point, it is essential to consider that in the initial part of the recording, we should have several seconds without any mouse to take this moment as our frame background. If you don’t have this option, you can use a photo or the software without the frame background (depending on the type of analysis) (Fig. 4A). After that, we selected the *initial frame* (when we had all mice in the corresponding arenas) and the *final frame*, which marks the start and the end of the evaluation period (Fig. 4B).
- In the *detection setting*, you can change different tools to improve mouse detection and also to mark the number of frames per second (FPS) of the recording (this information is usually available in the video settings) (Fig. 5).
- The final point is to select the *folder to save* and *run* the analyzes, which can take some minutes.
- After the initial analysis, we selected the *ROIs* (in this case, the central zone) and analyzed all the measures considering these zone differences (Fig. 1). After ROI selection, we did the *plots* and *Extras clustering* analysis of our data, as described in the Results section.

### Statistical analysis

Statistical analysis was performed using Student’s *t* test to compare both types of measurements in the LDB and OF tests with Jamovi 2.3.18 software.

## Results

This current study introduces novel and accessible software to evaluate rodent behavior. The software encompasses various aspects, such as motor activity and anxiety behaviors, and facilitates subsequent data analysis, including unsupervised clustering of mouse angles and trajectories. Its user-friendly design ensures ease of use, irrespective of the researcher’s programming skills, as no coding is necessary. The software produces reliable results, permitting further investigation into identifying similarities among groups of subjects through unsupervised clustering. Additionally, it aids in detecting outliers and serves as a valuable tool for result quality control. The results obtained from this study can be summarized as follows:

1. Validation of the ESTraS using the light–dark box (LDB) test.
2. Validation of the ESTraS using the open field (OF) test.
3. Unsupervised clustering by trajectories.
4. Unsupervised clustering by angles.

### Validation of the ESTraS using the LDB test

**Fig. 6:**
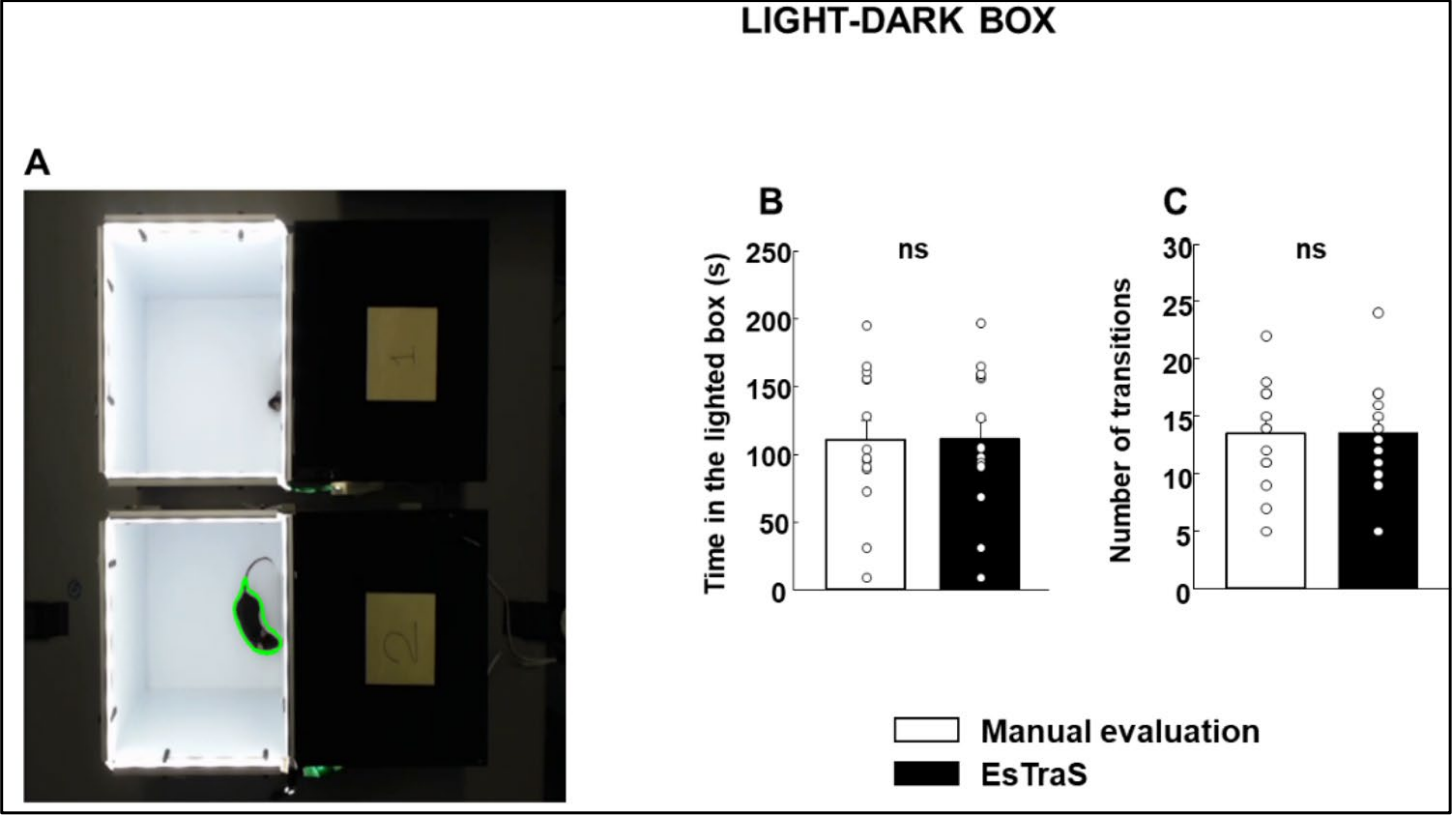
Validation of the ESTraS software using the light–dark box test (LDB) (**A**) in comparison with the manual quantification of the time in the lighted box (s) (**B**) and the number of transitions (**C**). No differences were observed between both types of evaluations. *N* = 14–15 animals/evaluation

In this study, and to validate ESTraS, a software designed for automated data analysis, a manual assessment was conducted to measure the time spent in the lighted box and the number of transitions (Fig. 6). Remarkably, there were no statistically significant differences observed between the manual measurements and ESTraS results for both parameters (Student’s *t* test; Fig. 6B: *t*(26) = – 0.00285, *P* = 0.998; Fig. 6C: *t*(26) = 0.000, *P* = 1.000).

### Validation of the ESTraS using the OF test

**Fig. 7:**
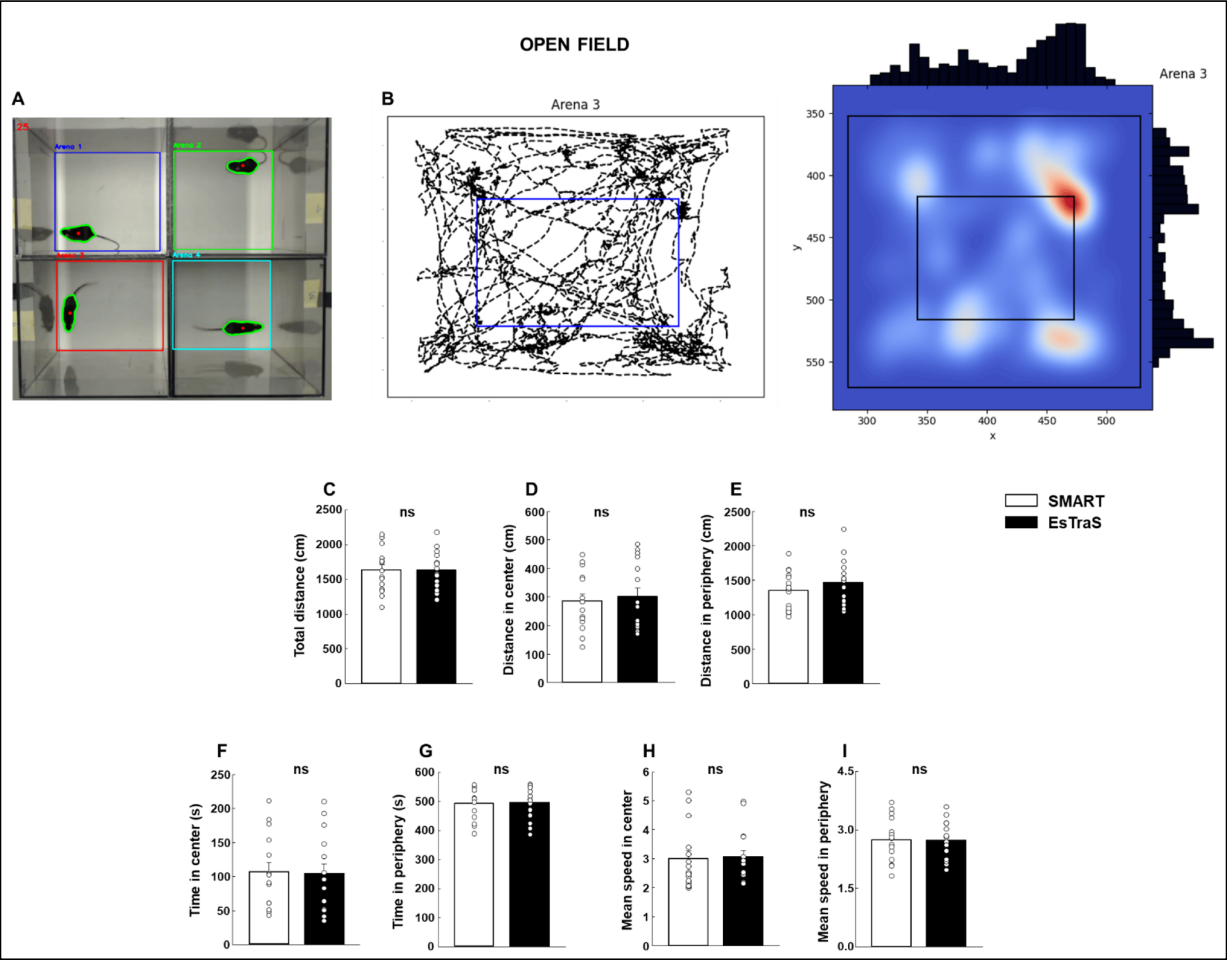
Compared to commercially available software, validating the ESTraS software using the open field test (OF) to assess rodents’ motor activity and anxiety-like behaviors (**A**). An example of the trajectory followed by a mouse in the arena and heatmap of the same trajectory (**B**). Total distance traveled (**C**), the distance in the center (**D**), and periphery (**E**), the time spent in the center (**F),** and periphery (**G**), as well as the mean speed in central (**H**) and peripheral (**I**) zones were evaluated. *N* = 15–16 animals/evaluation

We employed commercially available software for this test to validate the outcomes obtained from ESTraS. The parameters evaluated included the total distance traveled, the distance traveled in the center and periphery, the time spent in the central and peripheral zones, and the mean speed within each area. As illustrated in Fig. 7, no differences were observed between the results obtained from the SMART System and ESTraS across all the assessed parameters (Student’s *t* test; Fig. 7B: *t*(28) = 0.0179, *P* = 0.986; Fig. 7C: *t*(28) = – 0.106, *P* = 0.917; Fig. 7D: *t*(28) = 0.0629, *P* = 0.950; Fig. 7E: *t*(28) = 0.120, *P* = 0.905; Fig. 7F: *t*(28) = – 0.0596, *P* = 0.953; Fig. 7G: *t*(28) = – 0.178, *P* = 0.860; Fig. 7H: *t*(28) = 0.113, *P* = 0.911). This successful alignment of outcomes confirms the validity of the ESTraS software for use in conjunction with the OF test.

### Unsupervised clustering by trajectories

**Fig. 8:**
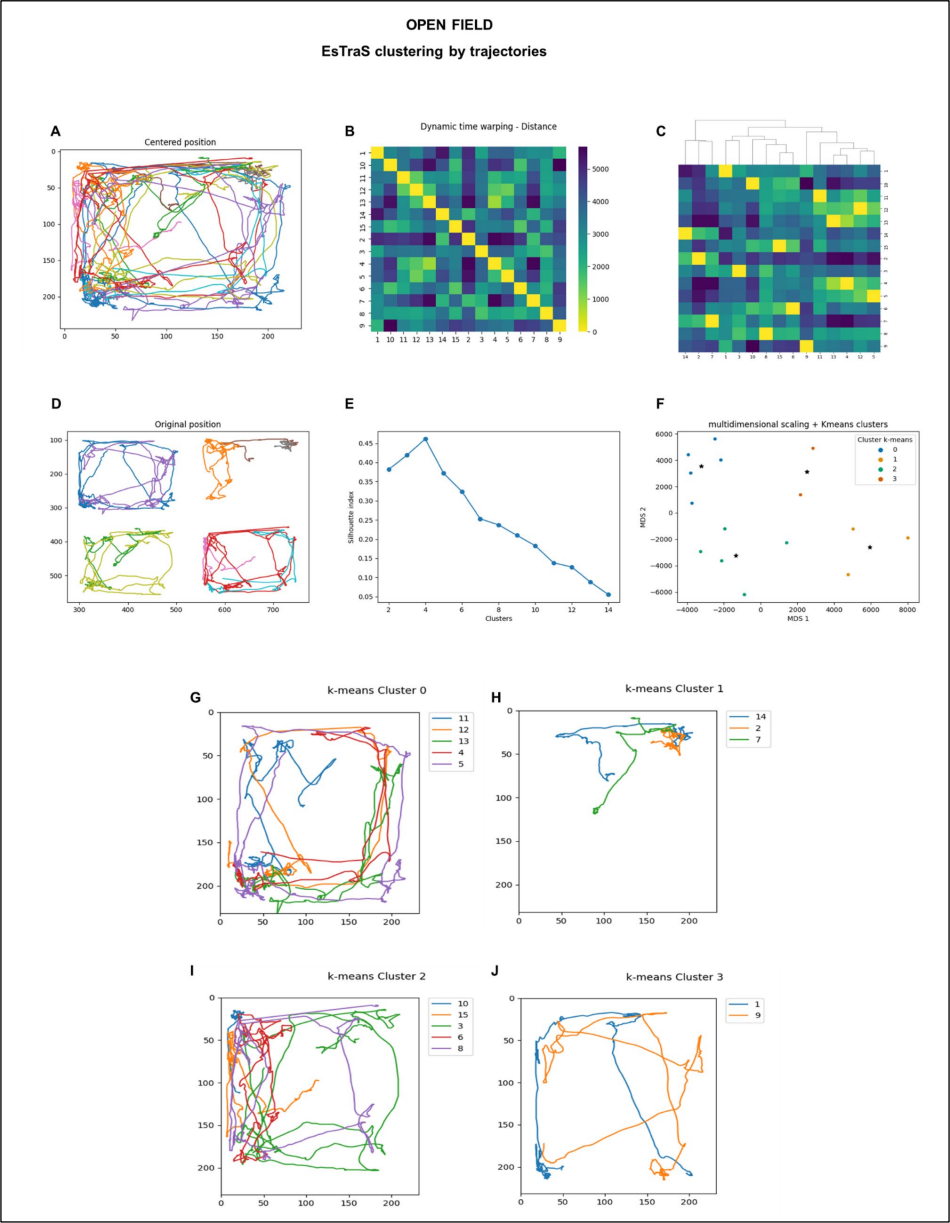
Unsupervised clustering by trajectories using the *k*-means method of the data obtained in the OF test. A sample of how ESTraS computes trajectories (**A**). A matrix array of dissimilarity distances is calculated by ESTraS (**B**). A heatmap of the distances clustered by dendrograms (**C**). The original positions of the trajectories before being adjusted to the same area (**D**). Selecting the number of clusters with silhouette analysis in *k*-means clustering (**E**). The figure represents distances using multidimensional scaling (MDS), with clusters generated by the* k*-means algorithm and their corresponding centroids (**F**). Each cluster’s trajectory is developed with the *k*-means algorithm (**G**, **H**, **I**, and **J**)

The software we developed distinguishes various trajectories adopted by mice after their placement in the center of the arena. In this study, we used this analysis specifically for the initial 30 s of the video-tracking process to observe the trajectories better (see Fig. 8). The software’s functionality involves providing trajectories based on the mice’s positions within the arena (A and D). By utilizing the dynamic time warping technique—a distance measure used for aligning and comparing sequences—similarities in the distance between all subjects were explored (B and C). During this stage, mice with significantly distinct trajectories were successfully identified (G, H, I, and J). To further investigate the data, we employed unsupervised clustering, enabling us to identify four distinct groups of mice (called cluster *k*-means 0, 1, 2, and 3). Corresponding centroids for each cluster (represented as E and F) were determined using the *k*-means method. This approach effectively categorized the mice based on their trajectory patterns and allowed for meaningful insights into their behavior.

### Unsupervised clustering by angles

**Fig. 9:**
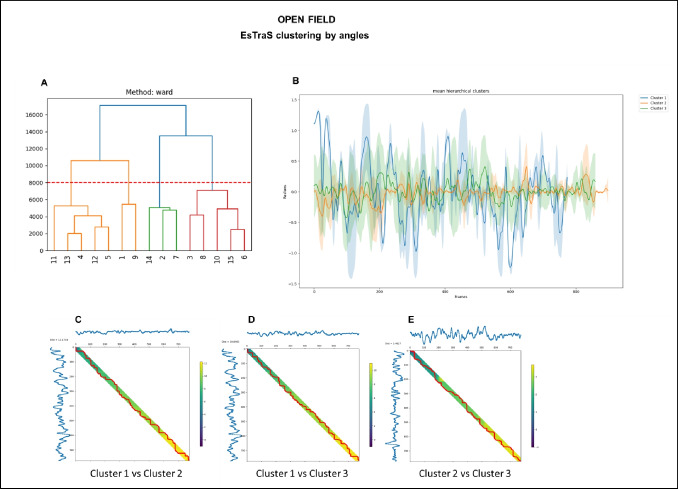
Unsupervised clustering by angles using the hierarchy method of the data obtained in the OF test. The dendrogram illustrates hierarchical clustering with a line indicating the cutoff point for generating clusters (A). The plot displays the angle mean of every cluster. Shaded bands surrounding each line indicate the standard deviation (B). A cost matrix represents the pairwise costs between two clusters (C, D, E)

Utilizing the results obtained from the OF test, we conducted an unsupervised clustering analysis based on angles, which provided additional insights into the composition of distinct groups among our subjects (Fig. 9). Employing the same data and evaluation time frame, we further investigated the angles adopted by mice using a different type of unsupervised clustering offered by ESTraS, namely the hierarchy clustering. The ward method was applied for this analysis, which aims to minimize within-group variance by iteratively merging two groups at each step of the hierarchy. It is worth noting that other available methods in ESTraS, including single linkage, complete linkage, average linkage, and centroid method, can also be employed per the researcher’s preference.

The application of the hierarchy clustering method led to the identification of four principal clusters (A). However, by setting the cutoff at 8000, we narrowed the analysis to three main clusters (B). We examined the mean angles and their corresponding standard deviations (B) for these three groups. The distinctions between these clusters were further evaluated through cumulative cost matrices, as shown in C, D, and E. These matrices facilitated comparisons of the angles adopted by mice from different clusters, effectively illustrating their similarities. Notably, a greater distance value indicated lower similarity, corroborating the findings presented in Fig. 9B.

**Fig. 10:**
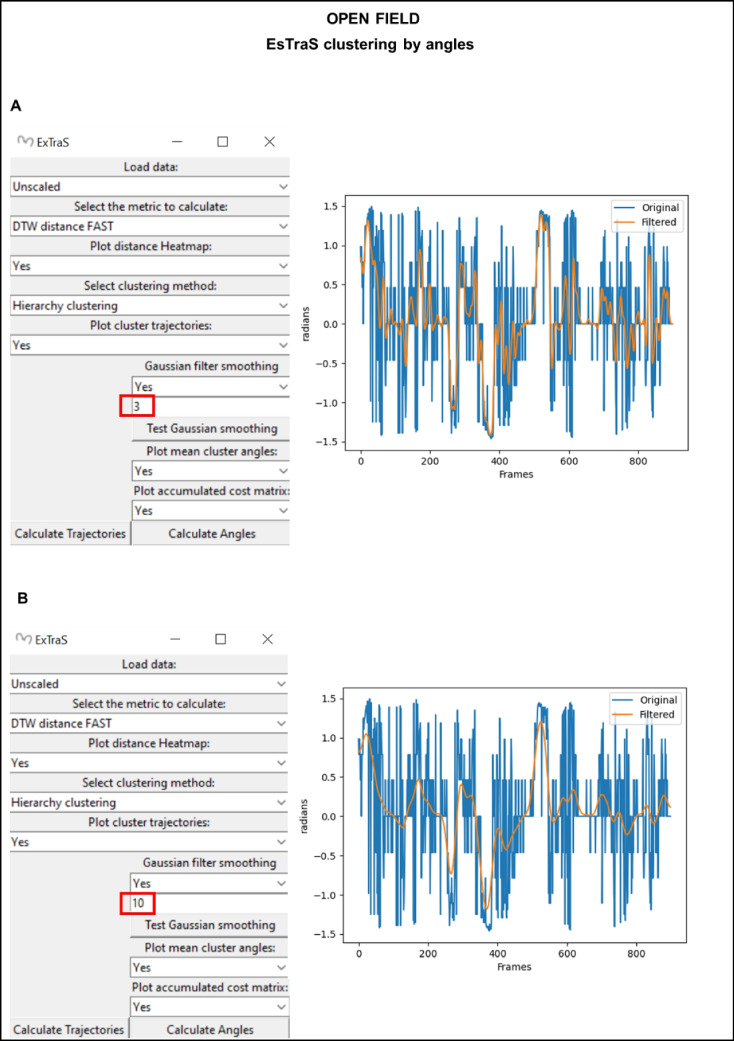
An example of applying a Gaussian filter with a kernel size of 3 (**A**) or 10 (**B**) to the original angles

To enhance the accuracy of the analysis, we implemented a Gaussian filter, as demonstrated in Fig. 10. The application of this filter proved to be a valuable tool in reducing angle noise, thus resulting in smoother representations of the mice’s detection (as seen in A and B of Fig. 10). The level of smoothness achieved varied depending on the filter applied, offering flexibility in adjusting the reduction of fluctuation noise. Overall, this comprehensive approach allowed us to gain valuable insights into the behavioral patterns of mice based on angle data, demonstrating the usefulness of the ESTraS software and the effectiveness of various analytical tools it provides.

## Discussion

The primary objective of this study was to validate the ESTraS software as an accessible and valuable tool for analyzing animal behavior, with a further exploration of the acquired data. The following results support this statement: (1) ESTraS demonstrated comparable outcomes to a manual evaluation in terms of the time spent in the lighted box and the number of transitions in the LDB test, and (2) The results obtained from the OF test were virtually indistinguishable between ESTraS and commercially available software, encompassing total distance traveled, distance traveled in ROIs, time spent in different ROIs, and mean speed in each ROI of the OF. ESTraS also facilitates data collection on the subjects’ trajectories and angles, enabling the classification of different subject groups using unsupervised clustering. Specifically, we demonstrated the utility of DTW followed by *k*-means or hierarchical clustering for this classification.

The freeware software for animal behavioral evaluation is gaining increasing importance in life sciences and research. However, most available tools require researchers to possess basic programming skills, and they often offer limited possibilities to explore the obtained results easily. In this context, ESTraS presents several crucial features that make it user-friendly and highly accessible. First, ESTraS is designed as a Python standalone executable, eliminating the need for researchers to install additional programs to work with it. Secondly, researchers can perform analyses and explore the results effortlessly with just a single click, without the necessity of programming skills. These aspects are significant and innovative, as most of the available open-source or freeware software typically demand programming knowledge or the installation of specific applications, thus requiring more substantial informatics resources (Pennington et al., 2019; Rao et al., 2019; Zhou et al., 2023).

Moreover, the software records subjects’ trajectories and angles, enabling unsupervised clustering using various methods based on their similarities, such as *k*-means and hierarchical clustering. ESTraS stands out as the first freeware software offering these advanced clustering options in one click. This feature is particularly intriguing and valuable for specific behavioral tests like object or social recognition. In these tests, how a rodent approaches an object or another subject can provide crucial information about its exploration skills and social behavior. By employing clustering tools, researchers can effectively detect subpopulations within the same group of subjects that may respond differently to the same stimulus. Furthermore, these tools can help distinguish between two populations clearly differentiated by the experimental manipulations applied, whether they are behavioral, pharmacological, or genetic.

In conclusion, the ESTraS software significantly advances animal behavioral analysis. Its user-friendly design, as a Python standalone executable, eliminates the need for researchers to possess programming skills or install additional programs. Including trajectory, angle recording, and unsupervised clustering options like *k*-means and hierarchical clustering sets ESTraS apart as pioneering freeware software.

## Data Availability

ESTraS is available at Ani Gasparyan’s GitHub page (https://github.com/A-Gasparyan/Easy-and-Simple-Tracking-System).

## Code availability

The code is under consideration for its intellectual protection at Miguel Hernandez University.
